# Coil combination of multichannel MRSI data at 7 T: MUSICAL

**DOI:** 10.1002/nbm.3019

**Published:** 2013-09-04

**Authors:** B Strasser, M Chmelik, S D Robinson, G Hangel, S Gruber, S Trattnig, W Bogner

**Affiliations:** a MR Center of Excellence, Department of Radiology, Medical University of ViennaVienna, Austria; b Athinoula A. Martinos Center of Biomedical Imaging, Department of Radiology, Massachusetts General Hospital, Harvard Medical SchoolBoston, MA, USA

**Keywords:** coil combination, phased array coil, MRSI, 7 T, noise correlation

## Abstract

The goal of this study was to evaluate a new method of combining multi-channel ^1^H MRSI data by direct use of a matching imaging scan as a reference, rather than computing sensitivity maps. Seven healthy volunteers were measured on a 7-T MR scanner using a head coil with a 32-channel array coil for receive-only and a volume coil for receive/transmit. The accuracy of prediction of the phase of the ^1^H MRSI data with a fast imaging pre-scan was investigated with the volume coil. The array coil ^1^H MRSI data were combined using matching imaging data as coil combination weights. The signal-to-noise ratio (SNR), spectral quality, metabolic map quality and Cramér–Rao lower bounds were then compared with the data obtained by two standard methods, i.e. using sensitivity maps and the first free induction decay (FID) data point. Additional noise decorrelation was performed to further optimize the SNR gain. The new combination method improved significantly the SNR (+29%), overall spectral quality and visual appearance of metabolic maps, and lowered the Cramér–Rao lower bounds (−34%), compared with the combination method based on the first FID data point. The results were similar to those obtained by the combination method using sensitivity maps, but the new method increased the SNR slightly (+1.7%), decreased the algorithm complexity, required no reference coil and pre-phased all spectra correctly prior to spectral processing. Noise decorrelation further increased the SNR by 13%. The proposed method is a fast, robust and simple way to improve the coil combination in ^1^H MRSI of the human brain at 7 T, and could be extended to other ^1^H MRSI techniques. © 2013 The Authors. *NMR in Biomedicine* published by John Wiley & Sons, Ltd.

## INTRODUCTION

^1^H MRSI enables the noninvasive investigation of local biochemical changes in healthy and pathologic brain tissue. However, as a result of the low metabolite concentrations, the signal-to-noise ratio (SNR) of *in vivo* MRS is intrinsically low. The SNR can be increased by the use of array coils (ACs), higher magnetic field strengths (e.g. 7 T) and shorter TEs.

With an efficient combination of the individual signals obtained from each channel, ACs provide a two to three times higher SNR than volume coils (VCs) 1,2, and enable accelerated data acquisition by the use of parallel imaging 4–5. However, AC data are challenging to combine whenever accurate phase information plays an important role, as in ^1^H MRSI 6 or phase imaging 7. The general coil combination uses complex weights *w_n_* to phase the spectra coherently and to weight spectra with higher SNR more heavily 8. Additional SNR can be gained by correcting for the noise correlation between the channels 1. The available methods for coil combination in ^1^H MRSI can be grouped into intrinsic and extrinsic reference methods.

Intrinsic reference methods estimate the complex weights *w_n_* from the acquisition under consideration itself, e.g. from the first free induction decay (FID) point (1stFIDpoint method) 9–10 by minimizing the difference between the magnitude and absorption spectrum 11, or based on the program LCModel 3.

Extrinsic reference methods, in contrast, use an additional reference scan to determine the combination weights *w_n_*. Two examples of such methods are the use of sensitivity maps (Sensmap method) 2 or an additional ^1^H MRSI scan without water suppression 12.

At higher field strength (e.g. 7 T), and for a larger number of channels, coil combination becomes increasingly challenging 7, but the potential increase in SNR and spectral resolution are significant. Recently, several approaches for ^1^H MRSI of the brain at 7 T have been proposed that promise to overcome the limitations caused by the high specific absorption rate, chemical shift displacement errors, shortened *T*_2_ times and increased *B*_0_/*B*_1_ inhomogeneities 13–21. Among these, the direct acquisition of the FID is one promising approach 13,14.

The established method for the ^1^H MRSI coil combination – using the first FID point – is problematic at 7 T and can lead to an incoherent data combination as a result of higher phase variations at higher magnetic fields, resulting in degraded spectral quality. The coil combination based on sensitivity maps, however, performs very well and allows the object-intrinsic phase component (e.g. iron deposition) to be preserved, but this phase component is irrelevant in ^1^H MRSI. In contrast, ^1^H MRSI requires the efficient elimination of all phase components that would interfere with an accurate quantification, including *B*_0_-induced, coil-specific and, also, anatomical phase variations. Only the phase component inherent to ideal FID oscillations and *k*-space encoding needs to be preserved. In addition, sensitivity maps cannot be estimated easily if no reference coil (i.e. body coil or VC) is available. With the advent of multi-transmit coils, particularly at higher field strength, body coils and VCs are frequently unavailable 22.

Therefore, we evaluated a new method for combining AC ^1^H MRSI data, which is based on the rapid acquisition of matching imaging calibration data within a few seconds. These imaging data are used directly as weights for coil combination, enabling a pre-phasing of spectra without any reference coil.

## THEORY

The general signal combination, as described by Roemer *et al*. 23, can be written as:


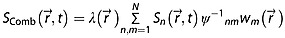
(1)

where 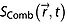 is the combined signal at position 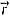 and time *t*, 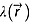 is the scaling factor; *N* is the number of channels; 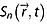 is the signal of channel *n*, 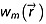 is the complex weighting of channel *m* and *ψ*_*nm*_ is the noise correlation between channels *n* and *m*. The latter can be computed from noise samples *χ* of size *N* × *k* by 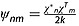, where *k* is the number of sampled points. For computational simplicity and clarity, a correlation-free AC is assumed in this theory part, i.e. *ψ*_*nm*_ = *δ*_*nm*_. The scaling factor 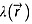 can be defined as:


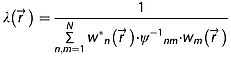
(2)

The factor *λ*, however, does not affect the SNR of the combined signal, but only rescales the combined data. This factor is crucial in order to eliminate the intensity profile of the AC if absolute metabolic concentrations are examined using external referencing. Yet, if only metabolic ratios are considered or absolute quantification is performed based on internal referencing, the scaling factor *λ* is of no importance.

The following equations describe the case when using an FID-based ^1^H MRSI sequence and a gradient echo (GRE) sequence with matching imaging parameters to combine the multichannel data. The proposed method is called ‘*Mu*ltichannel *S*pectroscopic Data Combined by Matching *I*mage *Cal*ibration Data’ (MUSICAL). MUSICAL can also be used with other sequences. The theoretical formulation might differ slightly in such cases. Let 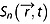 be the spectroscopic data and 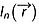 the imaging data of channel *n*, respectively, described by Equations [3] and [4].


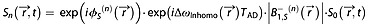
(3)


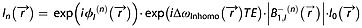
(4)

The first factors in both equations represent the channel-dependent phase, and the second factors represent the phases caused by different kinds of *B*_0_ inhomogeneity for an acquisition delay *T*_AD_ or TE. The third factors describe the magnitude reception profiles of coil *n*, and the final factors summarize channel-independent influences, such as relaxation, proton density, *B*_1_^+^ effects and water suppression.

The different coil combination methods mainly differ by the choice of the weighting factor *w_n_*. Three methods are described here: (i) the 1stFIDpoint method; (ii) the MUSICAL method; and (iii) the Sensmap method.

### 1stFIDpoint method

For the 1stFIDpoint method, the complex weights are chosen by:


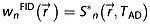
(5)

Combining Equations 1, 3 and 5 yields:


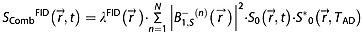
(6)

If the first FID point reflects the phase of the water resonance well, all channels are perfectly phased to zero before coil combination [i.e. all zero-order phase terms are eliminated and 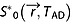 cancels the zero-order phase of 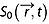 ]. However, this may not always be the case. The extent to which this holds is considered in the Discussion section.

### MUSICAL method

For the MUSICAL method, the weights are defined by:


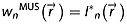
(7)

leading to:


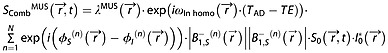
(8)

when combining Equations 1, 3, 4 and 7. The channel-dependent phase 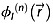 and magnitude 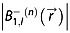 of the imaging data can be considered to be more accurate estimates of the *B*_1_^−^ field than those of the first FID point as a result of the higher SNR and the smaller influence of the fat signal (see Discussion section). Thus, the MUSICAL coil combination should outperform the 1stFIDpoint method, and the metabolite signal comprises no coil-specific phase after coil combination, even if *φ*_*S*_^(*n*)^ ≠ *φ*_*I*_^(*n*)^. This is no contradiction, when considering that *φ*_*S*_^(*n*)^ can be influenced by the fat signal, which usually has a different phase from the metabolite signals. If an imaging sequence similar to the MRSI sequence [
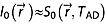
, except for a lower signal of the MRSI data as a result of water suppression] and TE = *T*_AD_ are chosen, the phase of the imaging data cancels the phase of the MRSI data, resulting in a combined signal that is phased to zero. No further phasing during spectral processing is then required.

### Sensmap method

Sensitivity maps can be computed by dividing the data of each channel by the data of a reference coil. The weights are defined as:


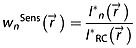
(9)

This leads to:


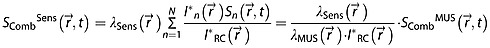
(10)

when combining Equations 1, 8 and 9. It is obvious from Equation 10 that the coil combination itself is equivalent to that of the MUSICAL method, only with a different scaling, leading to a phasing of the data based on the reference coil after coil combination. This method is ideal for combining imaging data, as any AC-specific phase information is removed, whereas the phase of the reference coil is introduced to the data and phase changes caused by anatomical *B*_0_ inhomogeneities are preserved. This is clearly not helpful in MRSI. Quite the contrary, it introduces an additional source of error, as the MRSI signal has to be later phased to zero before spectral processing.

## METHODS

### Subjects and hardware

Seven healthy volunteers were measured on a 7-T whole-body MR scanner (Magnetom, Siemens Healthcare, Erlangen, Germany) with a 32-channel AC for signal reception and a VC for signal reception/transmission (Nova Medical, Wilmington, MA, USA). One measurement was excluded as a result of motion artifacts. The remaining six datasets were processed further. Institutional Review Board approval was obtained. Written informed consent was obtained from all volunteers.

### Data acquisition

A three-dimensional *T*_1_-weighted magnetization-prepared rapid gradient echo (MPRAGE) sequence was acquired as an anatomical reference and for the creation of a brain mask. The sequence parameters were as follows: TE = 3.41 ms; TR = 3 s; TI = 1.7 s; GRAPPA factor of 3; matrix size, 256 × 246 × 160; nominal voxel size, 0.90 × 0.93 × 1 mm^3^. A *B*_1_^+^ map was acquired with a pre-saturation turboFLASH-based *B*_1_ mapping 24–25 sequence to calibrate the optimal pulse reference amplitude for the ^1^H MRSI slice under investigation.

The ^1^H MRSI data were acquired using a two-dimensional FID-based sequence 13 with 64 × 64 voxels (elliptically weighted *k* space acquired in a pseudo-spiral pattern), field of view (FoV) of 220 × 220 mm^2^, nominal voxel size of 3.4 × 3.4 × 12 mm^3^, *T*_AD_ = 1.3 ms, TR = 600 ms, spectral bandwidth of 6 kHz, 2048 complex FID points and weak WET water suppression 26. The acquisition time was 30 min. One volunteer was measured with and without water suppression. The non-water-suppressed data were acquired with the same parameters, except for water suppression and TR = 370 ms, leading to an acquisition time of 18.5 min.

A pair of GRE images with imaging parameters matching those of the ^1^H MRSI sequence, i.e. same matrix size, FoV, slice thickness, and pulse shape and duration, was acquired as a calibration scan for the coil combination. One of the GRE images was acquired with reversed imaging gradients to allow for the correction of minor phases introduced by the readout gradient. Other sequence parameters were TE = 1.3 ms, readout bandwidth of 1950 Hz/pixel and TR = 4 ms, resulting in a measurement time of 0.6 s. The TE of the GRE images matched the *T*_AD_ of the ^1^H MRSI sequence to ensure the same phase evolution [see Equation 8].

Volunteer 1 was measured with the VC to test whether the coil combination methods based on LCModel phase estimations are reliable for low-quality spectra. Volunteers 2–4 were measured with both AC and VC in the same session, whereas volunteer 5 was measured with only AC because of the long measurement time. Volunteer 6 was measured with a non-water-suppressed MRSI sequence in addition to the water-suppressed sequence to test whether the proposed coil combination method leads to comparable results to the ideal method. The overall measurement time was 1.5 h when measuring with both coils and 1 h when measuring with one coil.

### Pre-processing

Brain masks were obtained using the brain extraction tool BET2 (http://www.fmrib.ox.ac.uk/analysis/research/bet/) employing *T*_1_-weighted images. Only ^1^H MRSI voxels within this brain mask were processed.

Gradient delays during the acquisition of the GRE images caused a very minor linear phase gradient in the frequency-encoding direction. This phase gradient was eliminated by adding the complex data of normal GRE images to such images with reversed readout gradients. A FoV-dependent phase was added and elliptical filtering was performed to match the phase and point spread function of the ^1^H MRSI data, respectively.

The ^1^H MRSI data of all channels were combined using the GRE images [MUSICAL method, see Equation 8], the first FID point [1stFIDpoint method, see Equation 6], the first FID point of the non-water-suppressed MRSI data and the sensitivity maps [Sensmap method, see Equation 10] as complex weights. The scaling factor *λ* was computed according to Equation 2. The ^1^H MRSI data were Hamming filtered after coil combination in all approaches.

### Post-processing

After coil combination, the ^1^H MRSI data were fitted with LCModel (http://s-provencher.com/pages/lcmodel.shtml). The basis set was simulated using NMR scope from the jMRUI package (http://www.mrui.uab.es/mrui/mrui_Overview.shtml). The spectra have a first-order phase error caused by the acquisition delay. This error was taken into account by introducing the same error to the basis set by truncating the appropriate number of points at the beginning of the basis set FIDs 13–14. LCModel was not restricted in computing the zero-order phase when processing data with the Sensmap method or VC data, but was restricted to 0 ± 20° for the 1stFIDpoint and MUSICAL methods, leading to a faster spectral fitting. After LCModel fitting, the results were processed using MATLAB (MathWorks, Natick, MA, USA), and metabolic, phase and Cramér–Rao lower bounds (CRLB) maps were created. The SNR, defined as the signal of *N*-acetylaspartate (NAA) divided by twice the standard deviation of the noise in the frequency domain, was also calculated in MATLAB.

### Evaluation

#### Feasibility of the MUSICAL method

Before implementing the new coil combination algorithm, the consistency between the phases obtained from images and from ^1^H MRSI data was evaluated from the scans of four volunteers, which were acquired with VC. This was necessary to evaluate whether the imaging data can provide an accurate prediction of the ^1^H MRSI phase, and thus provide good coil combination weights. The phase of the ^1^H MRSI data was estimated in two ways: using LCModel and the first complex FID point. Both ^1^H MRSI phase maps were compared voxel-wise with the imaging phase maps.

The feasibility of the MUSICAL method in conjunction with the FID-based approach was further tested by computing the amount of voxels with CRLBs < 20% for the low-signal metabolites glutathione, *N*-acetylaspartyl glutamate (NAAG), aspartate, γ-aminobutyric acid (GABA) and taurine (Tau). A sample spectrum combined with the MUSICAL method and with a corrected first-order phase is provided in comparison with the same spectrum without the correction.

#### Phase estimation by LCModel

Forty voxels with high spectral quality (SNR > 17 and linewidth < 12 Hz) were selected from one VC dataset. Based on these spectra, different SNRs in the range 1–20 and linewidths in the range 8–37 Hz were simulated by adding white noise or by apodizing the FID and adding white noise to compensate for the increased SNR, respectively. The resulting datasets were processed with LCModel without any restriction in its phase computation. The phase deviation to the unmodified spectra, as a function of the simulated SNR and linewidth, was evaluated to show the variability of estimating coil combination weights with LCModel, as proposed by Maril and Lenkinski 3.

#### Comparison with other methods

The performance of the MUSICAL method was compared with that of the two standard methods, i.e. the 1stFIDpoint method and the Sensmap method, for the datasets of five subjects, and with the first FID point of the non-water-suppressed MRSI data in the case of volunteer 6. The appearance of problematic spectra near the skull, metabolic ratio maps, CRLB values and SNR were quantitatively and qualitatively compared between the three methods.

#### Noise decorrelation

The SNR improvement when performing noise decorrelation between the AC channels was evaluated, as suggested by Wright and Wald 1. The noise correlation matrix *ψ*_*nm*_ was computed from the last 200 FID points of voxels outside the head, and taken into account during coil combination with the MUSICAL method, according to Equation 1. For volunteer 5, additional noise-only data were measured with an FID sequence without any localization or radiofrequency pulses to test whether voxels outside the brain are a reliable source of noise data. The SNR was compared with and without noise decorrelation for volunteers 2–6, and for volunteer 5 also between the two different sources of noise.

## RESULTS

### Feasibility of the MUSICAL method

**Figure 1:**
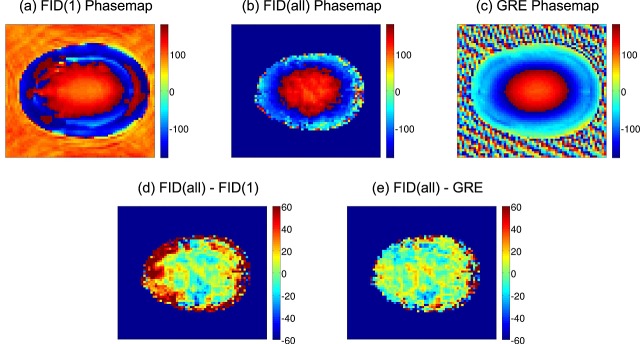
Comparison of the phase maps of volunteer 2 (in degree) computed from: (a) the first free induction decay (FID) point of the ^1^H MRSI data; (b) all FID points by LCModel; (c) gradient echo (GRE) imaging data. Subtraction maps are illustrated: (d) FID(all) − FID(1); (e) FID(all) − GRE. The FID(all) and GRE phase maps agree better than the FID(1) and FID(all) phase maps, suggesting that the GRE data provide a better phase estimation than the first FID point.

In Fig. 1, the phase maps computed using the first FID point [FID^1^] (Fig. 1a), the whole FID by LCModel [FID(all)] (Fig. 1b), the GRE data (Fig. 1c) and the subtraction phase maps, FID(all) − FID^1^ (Fig. 1d) and FID(all) − GRE (Fig. 1e) are shown for one volunteer. The phase differences are listed in Table 1 for volunteers 1–4. The phase of the GRE data matched that of the ^1^H MRSI data quite well after correction for gradient delays and FoV-dependent phase offsets (see Fig. 1 and Table 1). These data also indicate that the FID(all) and GRE phases agreed better than the FID(all) and FID^1^ phases, as the standard deviations of the latter subtraction maps were higher (*p* < 0.05 with paired *t*-test using the standard deviations of volunteers 1–4). If the phase computed by LCModel is considered to be correct, which is an acceptable assumption for VC data for which the SNR is reasonable in the whole brain, the GRE phases will be better estimates than those computed by the first FID point.

**Table 1:** Means and standard deviations (SD) of the phase differences ‘FID(all) – GRE’ and ‘FID(all) − FID(1)’ within the brain mask of volunteers 1–4

Measurement	Phase FID(all) − GRE (mean ± SD) (deg)	Phase FID(all) − FID(1) (mean ± SD) (deg)
Volunteer 1	3.7 ± 33.7	7.4 ± 44.4
Volunteer 2	1.8 ± 31.5	17.9 ± 42.0
Volunteer 3	−3.5 ± 21.0	−15.8 ± 22.8
Volunteer 4	7.2 ± 16.8	10.1 ± 29.1

FID(all), computed using the whole free induction decay (FID) by LCModel; FID(1), computed using the first FID point; GRE, computed using the gradient echo data.

**Figure 2:**
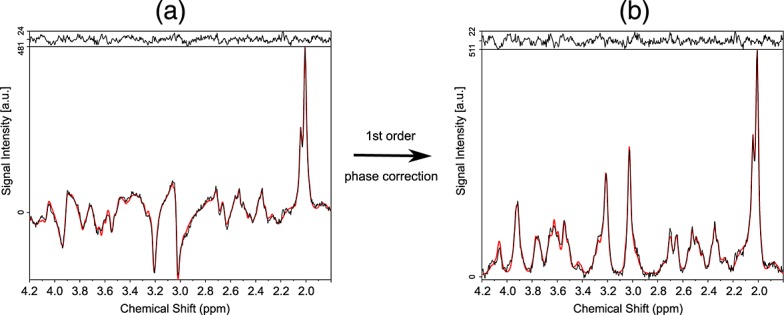
High-quality spectrum processed with the *Mu*ltichannel *S*pectroscopic Data Combined by Matching *I*mage *Cal*ibration Data (MUSICAL) method and fitted with LCModel. At the top of both spectra, the residuum, i.e. the difference between the measured spectrum (black line) and its fit (red line), is shown. The spectrum in (a) was not corrected for the first-order phase, whereas the spectrum in (b) was. In both cases, the baseline was subtracted. This spectrum shows the high quality of the spectra measured with a free induction decay (FID)-based sequence at 7 T and processed with the proposed method.

The percentages of brain voxels with CRLBs < 20% were 93.0%, 84.3%, 32.7%, 78.6% and 79.9% for the metabolites glutathione, NAAG, aspartate, GABA and Tau, respectively, when processed with MUSICAL and pooled over all volunteers. A first- and zero-order phase-corrected high-quality spectrum processed with the MUSICAL method is shown in Fig. 2.

### Phase estimation by LCModel

**Figure 3:**
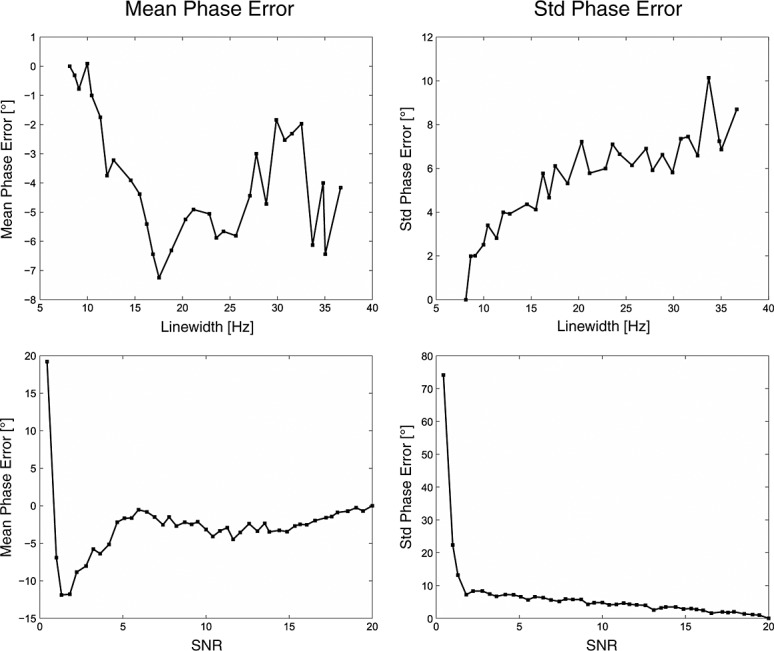
The means and standard deviations (SD) of the phase error (in degree) dependent on the linewidth (top) and the signal-to-noise ratio (SNR) (bottom). The phase varied highly with the SNR and linewidth. These graphs show that the phase estimation of LCModel is not reliable at low SNRs or high linewidths.

The dependence of the phase estimation error on the simulated SNR and linewidth is shown in Fig. 3. The phase estimation varied strongly with the SNR and linewidth, although Gaussian noise and fast signal decay cannot influence the phase. This suggests that the LCModel phase estimation is not reliable for low SNRs or broad linewidths.

### Comparison of coil combination methods

**Figure 4:**
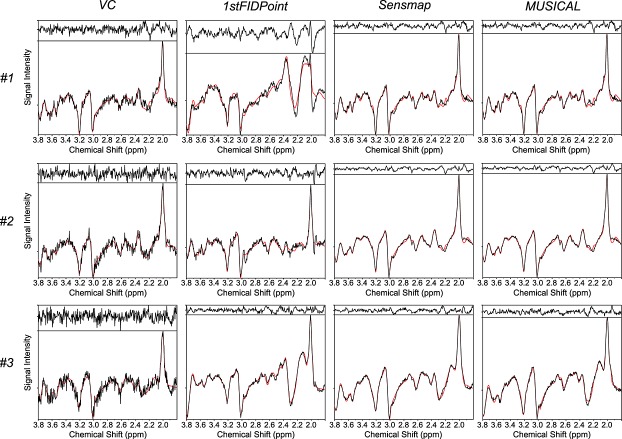
The LCModel results of three spectra for the volume coil (VC) measurement and the different array coil (AC) combination methods *Mu*ltichannel *S*pectroscopic Data Combined by Matching *I*mage *Cal*ibration Data (MUSICAL), Sensmap and 1stFIDpoint. At the top of each subimage, the residuum, i.e. the difference between the measured spectrum (black line) and its fit (red line), is shown. Spectrum #1 gives an example in which the 1stFIDpoint combination led to severe artifacts, whereas spectrum #2 provides an example in which the SNR was degraded strongly, but otherwise no artifacts occurred. Spectrum #3 shows that metabolic ratios might be altered [e.g. the glutamine and glutamate (Glx) peak at 2.3 ppm in comparison with the *N*-acetylaspartate (NAA) peak at 2.0 ppm] when using the 1stFIDpoint method, even if the spectrum looks reasonable. The spectra are not first order phased as a result of the free induction decay (FID)-based sequence and the fitting approach used.

In Fig. 4, three spectra at the border of the brain, where coil combination was most problematic, are shown for the three compared coil combination methods and VC as a reference. The spectra that were combined using the 1stFIDpoint method showed more artifacts, altered SNR and differing peak ratios compared with the other methods and with the VC results, whereas the spectra resulting from the MUSICAL and Sensmap methods were more similar to the VC results. As expected, the MUSICAL and Sensmap spectra looked very similar.

**Figure 5:**
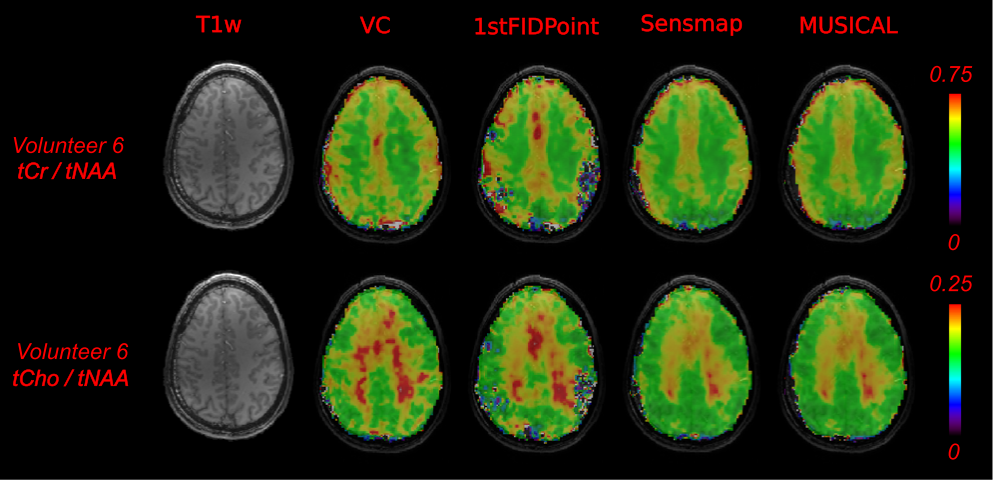
Comparison of metabolic ratio maps of the different coil combination methods and the volume coil (VC) measurement with a *T*_1_-weighted image of the same slice (T1w). Color-coded metabolic ratio maps were overlaid on *T*_1_-weighted images. There were no gross differences between the *Mu*ltichannel *S*pectroscopic Data Combined by Matching *I*mage *Cal*ibration Data (MUSICAL) and the Sensmap methods. The 1stFIDpoint method was problematic, especially at the border of the brain (colorless and purple voxels). All metabolic maps were interpolated from 64 × 64 to 128 × 128. tCho, total choline; tCr, total creatine; tNAA, total *N*-acetylaspartate.

In Fig. 5, the metabolic ratio maps of total Creatine (tCr) to total NAA (tNAA) [tCr/tNAA], and of total Choline (tCho) to tNAA [tCho/tNAA], are shown for one representative volunteer and all three coil combination methods and the VC measurement. This figure shows that, when using the 1stFIDpoint method, LCModel could not fit some of the brain regions, implying a suboptimal signal combination. In contrast, CRLBs > 20% were less numerous using the MUSICAL (9.2% of all CRLB values) or Sensmap (9.0% of all CRLB values) methods, compared with the 1stFIDpoint (17.6% of all CRLB values) method, for volunteers 2–6, when only the following metabolites were considered: GABA, myo-inositol (mI), Tau, tCho, tNAA, tCr, and glutamine and glutamate (Glx).

**Figure 6:**
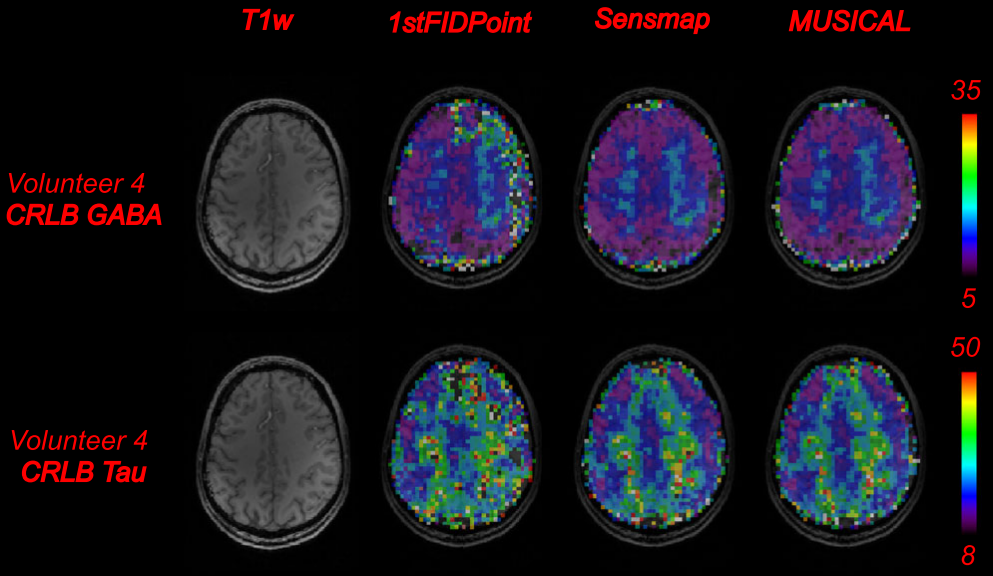
Comparison of Cramér–Rao lower bound (CRLB) maps of the metabolites γ-aminobutyric acid (GABA) and taurine (Tau) for the different coil combination methods for one volunteer. Color-coded CRLB maps were overlaid on anatomical *T*_1_-weighted images. Colorless voxels had CRLB values higher than 60 or LCModel could not fit these voxels. The 1stFIDpoint method showed more regions with higher CRLB values and more voxels with CRLB > 60.

In Fig. 6, CRLB maps of the brain metabolites GABA and Tau are shown for one volunteer and for the three coil combination methods. This figure shows, again, the equality of the MUSICAL and Sensmap methods, but illustrates that the 1stFIDpoint method leads to higher uncertainty in the fitting process. Table 2 shows the CRLB values of the brain metabolites GABA, mI, Tau, tCho, tNAA, tCr and Glx for all three coil combination methods. The CRLB values of the MUSICAL method were lower by 33.3% (*p* < 0.05 with paired *t*-test using the average CRLB values of all voxels and all aforementioned metabolites of volunteers 2–6), on average, than those of the 1stFIDpoint method, but were similar to those of the Sensmap method (*p* > 0.6 with paired *t*-test using the averaged CRLB values of volunteers 2–6). When using the additional non-water-suppressed MRSI data, the average CRLB values decreased by 1.5% for volunteer 6 in comparison with the MUSICAL method.

**Table 2:** Means and standard deviations (SD) of the Cramér–Rao lower bounds (CRLBs) of important brain metabolites for all three coil combination methods. All voxels within the brains of volunteers 2–6 were used to compute the means and SD. The means of the 1stFIDpoint method were substantially higher than those of the other two methods

Metabolite	1stFIDpoint (mean ± SD)	Sensmap (mean ± SD)	MUSICAL (mean ± SD)
GABA	17.5 ± 16.5	14.8 ± 14.6	14.8 ± 14.9
mI	10.0 ± 12.3	6.5 ± 8.0	6.7 ± 8.5
Tau	30.8 ± 16.6	22.4 ± 12.5	22.0 ± 12.2
tCho	8.9 ± 11.9	5.3 ± 7.0	5.4 ± 7.4
tNAA	6.1 ± 10.7	3.4 ± 5.6	3.5 ± 6.4
tCr	11.1 ± 16.0	6.3 ± 10.2	6.6 ± 10.9
Glx	7.8 ± 10.3	5.1 ± 4.7	5.1 ± 5.1

FID, free induction decay; GABA, γ-aminobutyric acid; Glx, glutamine and glutamate; mI, myo-inositol; MUSICAL, *Mu*ltichannel *S*pectroscopic Data Combined by Matching *I*mage *Cal*ibration Data; Tau, taurine; tCho, total choline; tCr, total creatine; tNAA, total *N*-acetylaspartate.

In Table 3, the mean and standard deviation of the SNR ratio between the three methods, evaluated within the brain, is listed for all AC datasets. These results demonstrate that the MUSICAL method performed better than the 1stFIDpoint method, not only in visual evaluation, but also led to a higher SNR, on average, by about 29.4% (*p* < 0.001 with paired *t*-test using the means of volunteers 2–6). The MUSICAL and Sensmap methods performed almost equally well, with slightly higher SNR values (1.7%) for the MUSICAL method (*p* < 0.01 with paired *t*-test using the means of volunteers 2–6). The SNR ratio between the MUSICAL method and that using the first FID point of the additional non-water-suppressed MRSI scan was SNR_MUS_/SNR_NonSupp_ = 1.02 for volunteer 6.

**Table 3:** Means and standard deviations (SD) of the signal-to-noise ratio (SNR) computed for the combination methods MUSICAL, Sensmap and 1stFIDpoint. All voxels within the brain masks were included

Volunteer	SNR 1stFIDpoint	SNR Sensmap	SNR MUSICAL
2	50.8 ± 18.3	67.4 ± 19.2	68.5 ± 19.2
3	58.5 ± 16.2	74.7 ± 17.3	76.0 ± 18.5
4	65.6 ± 22.9	87.9 ± 25.2	88.6 ± 26.0
5	62.6 ± 23.1	73.9 ± 19.1	76.3 ± 19.7
6	64.9 ± 14.7	80.8 ± 14.4	82.3 ± 13.6

FID, free induction decay; MUSICAL, *Mu*ltichannel *S*pectroscopic Data Combined by Matching *I*mage *Cal*ibration Data.

### Noise decorrelation

The SNR ratio with and without noise decorrelation is shown in Table 4 for volunteers 2–6. The average SNR increase for the four volunteers was 13.1 ± 2.0% (*p* < 0.001 with paired *t*-test using the means of volunteers 2–6). The use of additionally measured noise data rather than the end of FIDs outside the head did not change the resulting SNR of volunteer 5. Taking the noise correlation between the AC channels into account led to a significantly increased SNR, independent of which data were used to compute the noise correlation matrix *ψ*_*nm*_.

**Table 4:** Means and standard deviations (SD) of the signal-to-noise ratio (SNR) with (center) and without (right) noise decorrelation

Volunteer	SNR without noise decorrelation	SNR with noise decorrelation
2	68.5 ± 19.2	79.7 ± 25.1
3	76.0 ± 18.5	86.3 ± 22.5
4	88.6 ± 26.0	99.2 ± 30.6
5	76.3 ± 19.7	85.3 ± 22.0
6	82.3 ± 13.6	91.9 ± 16.3

## DISCUSSION

In this work, we have demonstrated a new method for combining multi-channel ^1^H MRSI data at 7 T. We provide evidence that the new method leads to better results than the most commonly used method, i.e. the 1stFIDpoint method, and to results similar to those of the Sensmap method. The proposed method increases the SNR, is computationally less demanding than the latter, needs no reference coil and results in phased spectra, making phase corrections during post-processing obsolete.

### Feasibility of the MUSICAL method

Imaging data with parameters matching those of the ^1^H MRSI data provided good estimates of the ^1^H MRSI phases, demonstrating the feasibility of the MUSICAL method. The first-order phase-corrected spectrum and the evaluation of low-signal metabolites showed that the MUSICAL method, in combination with a FID-based sequence at 7 T, can provide excellent spectral quality.

### Phase estimation by LCModel

Our data suggest that phase estimation by LCModel is unreliable for low spectral quality. Therefore, coil combination using LCModel phase estimates 3 is suboptimal for large coil arrays and in regions distant to individual elements (e.g. center of the brain). This can result in the incoherent summing of spectra, causing spectra with degraded quality and SNR.

### Comparison of coil combination methods

The 1stFIDpoint method is the most commonly used intrinsic reference method for MRSI, and is implemented on most scanners. It has the advantages of being fully automatic and computationally very simple, and results in reasonably well pre-phased combined spectra. However, in our study, its performance was worse than that of the MUSICAL method, when using a FID-based sequence at 7 T. The reasons for this are probably related to water suppression, lipid contamination and distorted water peaks. If water suppression is good, the magnitude of the first FID point is substantially reduced, thus increasing the uncertainty for estimating the coil weights *w_n_*, as shown by Dong and Peterson 10. In addition, close to the skull, the lipid contamination for different channels is affected by the individual coil channel sensitivity, leading to a local difference in residual water-to-fat ratio for each channel. Fat and water resonate at different frequencies. Hence, different phases are detected. The first FID point reflects a mixture of these phases. Thus, the 1stFIDpoint method can fail to sum spectra from lipid-contaminated voxels coherently. Moreover, if the residual water peak is distorted, this can cause additional phase problems when using the first FID point. In contrast, the water signals in the reference data of the MUSICAL and Sensmap methods are not suppressed, which is why contamination and other artifacts have little impact on the phases obtained.

Dong and Peterson 10 extended the 1stFIDpoint method by acquiring the ^1^H MRSI data without water suppression. This solves all the above-mentioned problems, but introduces problems with sideband artifacts at short TEs. Thus, Dong and Peterson 10 recommend the use of their method only at long TEs, which is unfavorable at 7 T.

Intrinsic reference methods performed in the spectral domain have been proposed by Prock *et al*. 11 and Maril and Lenkinski 3. Prock *et al*. 11 estimated the phase of the weights by minimizing the difference between the real and the magnitude spectrum within a specified spectral range near a major metabolite resonance. Maril and Lenkinski 3 estimated the complex weights using LCModel. Both methods are problematic when dealing with spectra of low SNR, e.g. far away from a coil's main sensitivity area, as the metabolite signal is highly affected by noise. In this study, we have shown that the phase estimation of LCModel is unreliable for low SNRs or high linewidths. Coil combination using LCModel for phasing is time consuming and cannot be implemented directly on a scanner.

The Sensmap method is the most important of the extrinsic reference methods. It is the best available coil combination method for imaging data 1–23, but is rarely used in MRSI coil combination. With this method, meaningful object-intrinsic phase information is preserved, the weights used are very insensitive to noise and the sensitivity maps can also be used for sensitivity encoding (SENSE)-based parallel imaging. However, the preservation of phase information is not necessary in MRSI. Indeed, in MRSI, any object/coil-dependent phase component that may bias accurate quantification should be removed. The Sensmap method introduces the phase of the reference coil, which is an additional error source for MRSI quantification. In addition, the necessary reference coil data cannot be acquired and can only be estimated if no VC or body coil is available 27.

A different extrinsic reference method determines the weights *w_n_* from an additional similar MRS(I) acquisition without water suppression 12–16. This method has no severe limitations other than the prolonged measurement time. The results in this study suggest no benefit of this method in comparison with the MUSICAL method.

### Noise correlation

Most previous studies on coil combination have ignored the effects of noise correlation 2–23. Other studies have reported very diverse SNR gains when performing noise decorrelation, ranging from 0.5% 28 to 40% 1, and up to 70% 29. In our study, we achieved an SNR increase of about 13%, which is slightly higher than that predicted by Roemer *et al*. 23, but much lower than the results of Wright and Wald 1 and Qian *et al*. 29. The most likely explanation for this large variation in SNR gain is the substantial difference in coil design. Although the gain achieved is not huge, our results underline the importance of noise decorrelation to optimize the SNR gain, particularly as it is easy to implement and can aid in the improvement of SNR-problematic MRSI scans.

### Performance at 7 T and other MRSI sequences

The MUSICAL method has been shown to perform well at 7 T and with FID-based sequences, which is most challenging as the phase is spatially more variable at higher field strengths, and the severe first-order phase poses additional difficulties. As a consequence, more care needs to be taken to avoid incoherent signal combination.

Our coil combination method could take ^1^H MRSI at 7 T further towards clinical practice, as it improves the SNR per unit time. By trading off the extra SNR against the sequence duration, e.g. with parallel imaging, the measurement time can be reduced significantly 4–5. Parallel imaging has been shown to perform even better at higher field strengths 30. Moreover, as two spatial dimensions are available for acceleration, high reduction factors can be expected.

In principle, the MUSICAL method can be extended to any type of MRSI sequence. For standard phase-encoded MRSI sequences, a matching imaging sequence can be achieved by omitting spectral acquisition and replacing one phase-encoding direction with frequency encoding. In fast MRSI sequences, such as spiral sampling, a faster matching imaging sequence can be achieved by omitting spectral data acquisition. The TR can be reduced to save time. It is important that the matching imaging sequence mimics the first FID point of the MRSI data as closely as possible, except for water suppression, with the same point spread function and the same phase evolution. The pulses of the MRSI and reference sequence should be the same or at least of a similar type and duration. A spin echo MRI sequence with the same TE and the shortest possible TR can be used as a reference sequence for a conventional spin echo MRSI sequence.

### Limitations

One disadvantage of the proposed coil combination method, MUSICAL, is the introduction of an additional weighting to the ^1^H MRSI data by the weighting with the imaging data. This can be corrected for by defining the scaling factor *λ* so as to obtain the uniform noise weighting described in ref. 8. Yet, when only metabolic ratio maps are of interest, the computation of signal ratios cancels out all common factors introduced to both metabolite maps, including this weighting.

## CONCLUSION

Our results show that the MUSICAL ^1^H MRSI coil combination method has significant advantages at 7 T using an FID-based sequence compared with two state-of-the-art methods, i.e. the 1stFIDpoint and Sensmap methods. The benefits include an increase in SNR, a decrease in CRLB values and an improved metabolic map and spectral appearance compared with the use of the 1stFIDpoint method, and an increase in SNR and a decrease in computational and hardware demands, with a pre-phasing of the resultant spectra, compared with the use of the Sensmap method. The pre-phasing increases the fitting speed, accuracy and reproducibility. In addition to the SNR increase enabled by ACs in conjunction with the MUSICAL method, noise decorrelation further enhances the SNR by 13%. In combination, the MUSICAL method is therefore an ideal tool with which to optimize the results of multichannel MRSI data, independent of coil hardware limitations.

## Abbreviations

AC: array coil
CRLB: Cramér–Rao lower bound
FID: free induction decay
FoV: field of view
GABA: γ-aminobutyric acid
Glx: glutamine and glutamate
GRE: gradient echo
mI: myo-inositol
MPRAGE: magnetization-prepared rapid gradient echo
MUSICAL: Multichannel Spectroscopic Data Combined by Matching Image Calibration Data
NAAG: N-acetylaspartyl glutamate
SENSE: sensitivity encoding
SNR: signal-to-noise ratio
T_AD_: acquisition delay time
Tau: taurine
tCho: total choline
tCr: total creatine
(t)NAA: (total)N-acetylaspartate
VC: volume coil
